# Validity and Reliability of Questionnaire on Knowledge, Attitude and Dietary Practices Related to Colorectal Cancer

**DOI:** 10.21315/mjms2020.27.1.12

**Published:** 2020-02-27

**Authors:** Bachok Norsa’adah, Aisyahtun Rosdi Aminu, Zakaria Zaidi

**Affiliations:** 1 Unit of Biostatistics and Research Methodology, School of Medical Sciences, Universiti Sains Malaysia, Kubang Kerian, Kelantan, Malaysia; 2 Department of Surgery, School of Medical Sciences, Universiti Sains Malaysia, Kubang Kerian, Kelantan, Malaysia

**Keywords:** colorectal cancer, validity, reliability, knowledge, attitude, practice, diet, lifestyle, Malay

## Abstract

**Introduction:**

Colorectal cancer (CRC) is one of the leading cancers in Malaysia where new cases are increasing every year. The aim of this study was to test the reliability and validity of a newly developed questionnaire on knowledge, attitude and dietary practices (KAP) related to CRC for the Malay population.

**Methods:**

The respondents were conveniently selected among visitors attending an outpatient clinic in a tertiary hospital. We excluded those with any cancers, chronic diseases and those that were illiterate. The exploratory factor and reliability analyses were conducted.

**Results:**

A total of 108 respondents were recruited of which 67.7% were males and the mean age was 54.59 years (standard deviation 8.93). The Kaiser-Meyer-Olkin (KMO) measure of sampling adequacy values for KAP were 0.64, 0.66 and 0.67, respectively (*P* < 0.001). The 17 items of knowledge formed five domains with loading factors ranging from 0.54–0.89. The six items of attitude formed two domains with loading factors ranging from 0.64–0.80 and the 15 practices had four domains with loading factors ranging from 0.52–0.83. The total variances explained for each KAP were 61.02%, 56.41% and 53.12%, respectively. The internal consistency Cronbach alpha values on KAP were 0.61, 0.60 and 0.70, respectively.

**Conclusion:**

The final questionnaire is suitable for measuring KAP related to CRC among the Malay population.

## Introduction

Colorectal cancer (CRC) is ranked the third most common cancer in men and second most common cancer in women in most parts of the world (1). There were 13,683 new cases of CRC that had been reported in the Malaysian National Cancer Registry between the years of 2007 and 2011 (2). CRC was the most common cancer in Malaysia for males (16.3%) and the second among females (10.7%) after breast cancer (32.1%) (2). The mortality due to CRC is increasing and is considered the fourth leading cause of cancer death in the world (1). Globally, up to 945,000 new cases of CRC have been diagnosed every year (3). The reported incidence rates of CRC in both males and females increased for 27 of 51 cancer registries considered worldwide (4).

The improved socioeconomic status and acceptance of a westernized lifestyle in the population of developing Asian countries including Malaysia is associated with an increased incidence of CRC (5). Malaysia is undergoing an ageing of its population (6) with increasing affluence and an increased prevalence of risk factors for CRC, including obesity, smoking and a westernised diet (4). Approximately 94.3% of CRC cases in Malaysia occurred in people over the age of 40 years with the highest proportion of cases between 60 and 69 years (2).

CRC can be prevented by creating an awareness of the disease, its signs and symptoms, the risk factors and screening methods. There was a significant association between CRC knowledge and screening test uptake (7). The Malaysian population may have different risk factors compared to those of Western populations, especially considering lifestyle and diet. There is a need to improve knowledge about CRC and its risk factors so that people may take screening tests or recognise symptoms in order to diagnose CRC at an early stage. It is generally recognised that early detection of CRC leads to early treatment and a better chance of curing the disease. Thus, a study of knowledge, attitude and practices is important to measure the awareness and successful implementation of a preventive programme among our population. Therefore, this study aimed to validate a newly developed questionnaire that focused on knowledge, attitude and dietary intake related to CRC among the Malay population.

## Methods

The respondents of this study were the outpatients in a tertiary hospital, which is the largest hospital in north-eastern Malaysia that provides services to all levels of socio-economic groups. Respondents were chosen randomly and asked for written consent and answered the questionnaire by themselves. We included respondents over the age of 30 years and excluded those with any reported cancers, chronic diseases, mental illness and illiteracy. All respondents who participated in the research were informed about the research and purposes and signed a written consent.

The development of this questionnaire was done based on expert discussion and published articles (7–9). The expert consisted of two colorectal surgeons and an epidemiologist. All questions were reviewed for content, understanding and comprehension. The questionnaire was in the Malay language and comprised of socio-demographic data, knowledge of CRC, attitude and practices on dietary intake.

The knowledge section consisted of 27 close-ended statements with three possible answers: yes, no and don’t know. For positive questions, the yes answer was given a score of two, the no answer was given a zero score, and the don’t know answer was given a score of one. A reverse score was given for negative questions.

There were nine attitude statements with a response score ranging from one to ten, corresponding to a score of strongly disagree to strongly agree. A reversed score was given for a negative statement. There were six positive statements and three negative statements. Respondents were asked to choose the best response to the provided statement based on their personal opinion.

A list of questions about diet frequency was developed for 21 food intake that may increase the risk or protect a person from the development of CRC. The food frequency responses were six scales of never, every day, more than three times a week, one to two times a week, more than three times a month, one to two times a month with a score ranging from 0 to 5 for foods that may reduce the risk of developing CRC. An inverse score was given for foods that may increase the risk.

## Statistical Analysis

Statistical analysis was performed using SPSS (Statistical Package of Social Science) version 24. Data were double entered and checked for any missing data. A descriptive analysis was carried out to determine the frequency, percentage, mean and standard deviation of the variables. All numerical variables were checked for normality of distribution.

Exploratory factor analysis (EFA) was used as the statistical method to identify the inter-relationships that exist among a large set of questionnaires. It is exploratory in nature to search for a possible underlying structure in the questionnaires and how they are related. We applied the principal component method and varimax rotation to extract items into construct subscales called factors (10, 11). We considered factors with an Eigenvalue of greater than one. Data assumptions were checked including normality of distribution, linearity, outliers and multicollinearity (12).

The reliability was obtained following EFA by estimating the Cronbach alpha internal consistency and Guttman split-half coefficients and corrected item-total correlations (CITC). We randomly divided the data into two and then tested the inter-class correlation between the datasets. The loading factor was set at 0.40 and above, the CITC was greater than 0.3 and the Cronbach alpha was greater than 0.7 (7, 12). The level of significance was less than 0.05.

## Results

A total of 108 respondents were included in the analysis. The socio-demographic and clinical characteristics of the respondents are presented in Tables 1 and 2, respectively. All were of Malay ethnicity and had a mean age of 54.59 years (standard deviation 8.93). The majority were 46 to 55 years old (38.0%), male (67.6%), married (95.4%) and had a secondary school education (59.3%). Students and retirees had a higher percentage than other occupations and a monthly income ranging from 0 to 2,000 (72.2%).

Tables 2–4 show the loading factors for knowledge, attitude and dietary practices on CRC. The Kaiser-Meyer-Olkin (KMO) values for knowledge, attitude and diet were 0.64, 0.66 and 0.67, respectively, which were considered acceptable. All the Bartlett’s tests showed significant values of *P* < 0.001, indicating the adequacy of the data. All of the items had high communalities values (> 0.3), which indicated that the items fit well with the other items in their factor.

For the knowledge section, the 27 items were initially loaded into 12 factors. We removed 10 items because the factor loads were low (< 0.4). The extraction of 17 items of knowledge was forced into five factors using the Principal Component Analysis (PCA) method and the Varimax rotation method with Kaiser normalisation. All items were finally accepted as they loaded significantly into five components with loading factors of greater than 0.4. The total variance explained for these items was an acceptable level (61.02%). The five factors of knowledge identified from the factor analysis were named as follows:

- symptoms of CRC, composed of four items ranging from 0.54 to 0.86 accounting for 21.54% of the variance
- symptoms and prevention, consisted of four items, factor loading ranging from 0.53 to 0.85 accounting for 11.40% of the variance
- overview and screening test of CRC, consisted of three items ranging from 0.53 to 0.90 accounting for 10.45% of the variance
- protective factor, consisted of three items ranging from 0.72 to 0.76 accounting for 9.22% of the variance
- risk factor, consisted of three items ranging from 0.70 to 0.74 accounting for 8.42% of variance

For the attitude part, the nine items were initially loaded into seven factors. Three items were removed because the factor loadings were low (< 0.4). The total variance explained by these items was an acceptable level (56.41%). The two factors of attitude were labelled as:

- healthy lifestyle, consisting of three items with high factor loading (0.70–0.74) accounting for 34.42% of the variance and
- health-seeking attitude, involving three items with a high factor loading (0.64–0.80) accounting for 22.00% of the variance.

For the practice on diet, the 21 items were initially loaded into six factors. Six items had low loading factors (< 0.4) and thus, were deleted. The 15 items were forced into four factors, which was acceptable as the loading factors were satisfactory, and the total variance explained was 53.12%. The four factors of practice related to diet were named as:

- protein diet, consisted of five items (0.50–0.71) accounting for 21.52% of the variance
- hot and spicy, consisted of four items (0.56–0.86) accounting for 12.46% of the variance
- meat and salted, consisted of four items (0.53–0.78) accounting for 10.33% of the variance
- fish and vegetable, consisted of two items (0.73–0.83) accounting for 8.81% of the variance

Tables 5–7 show the reliability for knowledge, attitude and diet practices on CRC. The overall Cronbach’s alpha scores for internal reliability of knowledge, attitude, and practice on diet were 0.61, 0.60 and 0.70, respectively. The Cronbach’s alpha scores were as follows: the knowledge factors were 0.75, 0.67, 0.72, 0.63 and 0.53, respectively; the attitude factors were 0.57 and 0.60, respectively; and the diet factors were 0.58, 0.62, 0.64 and 0.67, respectively. The intra-class correlation coefficient was 0.70 (95% CI, 0.61–0.78) with a *P* < 0.001.

## Discussion

This study aimed to validate a new tool for CRC-related knowledge, attitudes and dietary practices among the Malay population. This 38-item questionnaire takes only 20 min to answer and can be used to measure awareness of CRC and the effectiveness of the health education programme. This questionnaire includes dietary practices that are important factors related to the development of CRC. It was created in the Malay language as most Malaysians use it as their mother tongue and have adapted to their culture and practices. The questionnaire was designed to be self-administered to avoid interviewer bias. It is also suitable for use in high-risk age and low-socio-economic groups. This could be demonstrated by the distribution of our samples.

This study conducted an EFA, which is a powerful multivariable analysis examining structure and relationship between variables, as well as constructing validity of the questionnaire (10–12). The construct validity is measured using the principal component method and varimax rotation as the statistical technique. The KMO of the items measured ranged from 0.64 to 0.67, which indicated that the sample was adequate to perform a satisfactory factor analysis (10). The KMO levels in our study were considered moderate (12). Furthermore, the total variances explained by the selected factors for knowledge, attitude and dietary practices were 61.02%, 56.41% and 53.12%, respectively. These were above the recommended level of at least 30%.

We selected groups of domains based on the recommendations of the cumulative percentage of variance and Eigen value of more than one. The original 27 knowledge, 9 attitudes and 21 dietary intakes were tested, and several items were eventually dropped in order to obtain valid satisfactory results. Several items were deleted due to a low loading factor (< 0.4). The items of knowledge, attitude and practice on diet finally loaded into five, two and four factors, respectively. We had a problem in naming our factors because some of the items did not fit into one group. It has been mentioned that naming the factors is subjective and reflects conceptual intention (10).

The most satisfactory overall Cronbach alpha score was for dietary practice, followed by knowledge and attitude. A detailed examination for each factor in each section of the questionnaires revealed that the internal consistency for symptoms and, overview and screening test factors, was good (> 0.7), moderate for other factors and lower for healthy lifestyle attitude (0.57) and knowledge regarding risk factors (0.53). We retained these factors with low reliability because they are important to include in the whole set of questions. A subsequent study is recommended to paraphrase these questions.

This study had several limitations. The responses from the respondents were inclined to recall bias especially for dietary intake. The sample size was relatively small. There is a lack of agreement and varying opinions on the required sample size (10, 12). However, we used a sample to variable ratio recommendation that a minimum three times for the number of items is adequate. The largest items in our study were 27, thus we need a minimum of 81 samples which was exceeding our numbers.

Furthermore, we examined the communality of each item. The communality represents the amount of variance accounted for by the factor solution for each item, that at least one-half of the variance of each item is considered (11). There were four items in the knowledge, one in attitude and five in dietary practices with communalities of less than 0.50. These were additional drawbacks of this study.

Many items were removed from the analysis to satisfy the minimum requirements. As a result, some questions about risk factors and dietary intake were not included in our final questionnaires. Another larger sample study is needed, with many additional items included in different phrasings. This study also needs to be repeated for different ethnicities and regions in Malaysia.

## Conclusion

The final questionnaire is a valid and reliable tool to measure knowledge, attitude and dietary practices related to CRC in the Malay population.

## Figures and Tables

**Table 1:** Socio-demography characteristics of respondents (*n* = 108)

Socio-demography of respondents		Frequency (%)
Age (year)		54.59 (8.93)*
	35–45	19 (17.6)
	46–55	41 (38.0)
	56–65	30 (27.8)
	66–75	18 (16.7)
Gender	Male	73 (67.6)
	Female	35 (32.4)
Marital status	Widowed	5 (4.6)
	Married	103 (95.4)
Level of education	Tertiary	26 (22.2)
	Secondary school	64 (59.3)
	Primary school	12 (11.1)
	Others	8 (7.4)
Occupation	Unemployed	26 (24.1)
	Government	23 (21.3)
	Private	2 (1.9)
	Self-employed	23 (21.3)
	Others	34 (31.5)
Monthly income (RM)		1497.84 (1726.06)*
	0–2,000	78 (72.2)
	2,001–4,000	23 (21.3)
	> 4,000	7 (6.5)

Note:

* mean (standard deviation)

**Table 2:** Loading factors of knowledge questionnaires on CRC

Items	Statements	Factors					Communalities

		1	2	3	4	5	
K40	Symptom of CRC includes passing blood with motion	0.54					0.46
K41	Symptom of CRC includes constipation	0.61					0.45
K43	Symptom of CRC includes loss of appetite	0.86					0.80
K44	Symptom of CRC includes weight loss	0.84					0.78
K26	CRC causes growth on bowel surface area		0.54				0.50
K28	CRC can be prevented		0.85				0.75
K29	CRC can be cured if treated early		0.80				0.70
K45	Symptom of CRC includes stomach-ache		0.53				0.48
K25	CRC is a cancer of the colon and rectum			0.53			0.43
K47	A screening test of CRC includes sigmoidoscopy			0.90			0.81
K48	A screening test of CRC includes colonoscopy			0.89			0.83
K33	Taking fish can increase the risk of CRC				0.72		0.56
K34	Taking soymilk can increase the risk of CRC				0.73		0.54
K35	Taking green vegetables can increase the risk of CRC				0.76		0.60
K36	Smokers have a high risk of CRC					0.74	0.60
K38	A family history of CRC increases the risk of CRC					0.70	0.58
K39	Obesity is a risk factor of CRC					0.70	0.52

Notes: Factor analysis; Extraction method: principal component analysis; Rotation method: Varimax with Kaiser normalisation; KMO was 0.64; Bartlett’s test of sphericity was significant ( *P* < 0.001), total variance explained was 61.02%

**Table 3:** Loading factors of attitude questionnaires on CRC

Items	Statements	Factors		Communalities

		1	2	
A52	I will take more green vegetables to prevent CRC	0.70		0.49
A53	I will take less meat to prevent CRC	0.74		0.55
A54	I will exercise to prevent CRC	0.73		0.54
A55	I am willing to do a screening test to detect CRC		0.64	0.56
A58	I will allow a doctor to examine my rectum		0.78	0.61
A60	I will consult a doctor urgently if I have a defecation problem		0.80	0.64

Notes: Factor analysis; Extraction method: principal component analysis; Rotation method: Varimax with Kaiser normalisation; KMO was 0.66; Bartlett’s test of sphericity was significant ( *P* < 0.001); total variance explained was 56.41%

**Table 4:** Loading factors of practice on diet questionnaires

Items	Diet	Factors				Communalities

		1	2	3	4	
P63	Chicken	0.71				0.52
P66	Egg	0.59				0.41
P72	Anchovy soy	0.55				0.56
P79	Coconut milk-based food	0.52				0.33
P64	Liver/gizzard of chicken	0.50				0.36
P80	Spicy food		0.86			0.76
P73	Shrimp paste		0.62			0.59
P68	Chilies		0.59			0.51
P81	Curry based food		0.56			0.41
P76	Salted fish			0.78		0.67
P75	Salted eggs			0.73		0.63
P62	Internal organs of animals			0.58		0.41
P61	Meat			0.53		0.53
P65	Fish				0.83	0.73
P67	Green vegetables				0.73	0.56

Notes: Factor analysis; Extraction method: principal component analysis; Rotation method: Varimax with Kaiser normalisation; KMO was 0.67; Bartlett’s test of sphericity was significant ( *P* < 0.001); total variance explained was 53.12%

**Table 5:** Reliability of knowledge questionnaires on CRC

Items	Factor	Cronbach’s alpha	Guttman split-half coefficient	Corrected item-total correlation	Cronbach’s alpha if item deleted
K40	Symptoms	0.75	0.59	0.42	0.75
K41				0.43	0.75
K43				0.70	0.60
K44				0.65	0.63
K26	Symptoms and prevention	0.67	0.66	0.31	0.74
K28				0.64	0.50
K29				0.57	0.56
K45				0.42	0.64
K25	Overview and screening test	0.72	0.73	0.33	0.92
K47				0.66	0.78
K48				0.68	0.46
K33	Protective factor	0.63	0.59	0.40	0.57
K34				0.47	0.49
K35				0.44	0.52
K36	Risk factor	0.53	0.48	0.39	0.36
K38				0.31	0.48
K39				0.33	0.45

Note: Overall Cronbach’s alpha value was 0.61

**Table 6:** Reliability of attitude questionnaires on CRC

Items	Factor	Cronbach’s alpha	Guttman split-half coefficient	Corrected item-total correlation	Cronbach’s alpha if item deleted
A52	Healthy lifestyle	0.57	0.52	0.37	0.49
A53				0.41	0.41
A54				0.37	0.49
A55	Health seeking attitude	0.60	0.53	0.38	0.53
A58				0.39	0.54
A60				0.48	0.44

Note: Overall Cronbach’s alpha value was 0.60

**Table 7:** Reliability of practices on diet questionnaires

Items	Factor	Cronbach’s alpha	Guttman split-half coefficient	Corrected item-total correlation	Cronbach’s alpha if item deleted
P63	Protein	0.58	0.48	0.44	0.46
P66				0.31	0.53
P72				0.27	0.59
P79				0.35	0.51
P64				0.35	0.51
P80	Hot & spicy	0.62	0.64	0.62	0.36
P73				0.35	0.58
P68				0.30	0.61
P81				0.35	0.60
P76	Meat & salted	0.64	0.65	0.40	0.59
P75				0.41	0.60
P62				0.53	0.50
P61				0.41	0.61
P65	Fish & vegetable	0.67	0.67	0.51	-
P67				0.51	-

Note: Overall Cronbach’s alpha value was 0.70
